# Repeated Short-Term Intratracheal Exposure to Urban Particulate Matter Has No Apparent Adverse Effects on Female Reproductive Outcomes in Mice

**DOI:** 10.3390/cells15171522

**Published:** 2026-08-24

**Authors:** Seo Hyeon Mun, Hyunsun Park, Doh Hee Kim, Jae Hyeok Heo, Myeong Jin Yeon, Sun-Hee Lee, Haengseok Song

**Affiliations:** 1 Department of Life Sciences, Graduate School, CHA University, Pocheon 11160, Republic of Korea; seohyeon9460@gmail.com (S.H.M.); hyunsun9510@naver.com (H.P.); 2 Department of Research Institute, Seoul Medical Center, Seoul 02053, Republic of Korea; dohheekim@seoulmc.or.kr (D.H.K.); drjae93@seoulmc.or.kr (J.H.H.); 3 Department of Neurology, Seoul Medical Center, Seoul 02053, Republic of Korea; 4 Seoul MC Fertility Center, Seoul Medical Center, Seoul 02053, Republic of Korea; mjyeon77@gmail.com (M.J.Y.); shlee1027@hotmail.com (S.-H.L.); 5 Division of Life Sciences, CHA University, Pocheon 11160, Republic of Korea

**Keywords:** urban particulate matter, polycyclic aromatic hydrocarbons, pulmonary inflammation, female reproduction, preimplantation embryo development

## Abstract

Urban particulate matter (UPM) is a major environmental pollutant known to induce systemic inflammation. While its respiratory toxicity is well-documented, the potential vulnerability of the female reproductive system to inhaled UPM remains a critical concern. In this study, we evaluated female reproductive outcomes in a murine model following repeated short-term intratracheal instillation of UPM (40–200 mg/kg) for a month. Our results demonstrate that while UPM exposure induced robust, dose-dependent pulmonary inflammation, characterized by immune cell infiltration (CD45+, F4/80+, and Ly6G+) and upregulation of pro-inflammatory (*Il-6*, *Il-1β*, and *Tnfα*) and fibrotic (*Col1a1* and *Tgfβ1*) markers, no apparent adverse effects were observed in the reproductive endpoints examined. No significant alterations were observed in maternal pregnancy outcomes, including oocyte recovery rates, fertilization competence, gestation duration, litter size, and postnatal development of pups. Furthermore, the mRNA expression of inflammatory markers in the ovaries and uteri was not detectable. Direct in vitro exposure of two-cell embryos to UPM did not interfere with preimplantation embryo development. Although benzanthracene slightly reduced the blastocyst formation rate, UPM and polycyclic aromatic hydrocarbons, major toxic components of UPM, did not severely compromise preimplantation embryo development in vitro. In conclusion, these findings suggest that marked UPM-induced pulmonary inflammation is not accompanied by substantial alterations in the female reproductive outcomes examined under short-term exposure conditions.

## 1. Introduction

Ambient air pollution, specifically particulate matter (PM), represents a formidable global health challenge linked to increased morbidity and mortality from diverse systemic pathologies [1,2]. As a major constituent of atmospheric pollution, PM is a complex mixture of solid and liquid particles that can penetrate deep into the respiratory system and has been reported to trigger systemic oxidative stress [2,3,4]. Within this context, urban particulate matter (UPM), as exemplified by the SRM 1648a standard, is a particularly hazardous heterogeneous mixture characterized by a high load of adsorbed toxic elements, including heavy metals and polycyclic aromatic hydrocarbons (PAHs) [5,6]. While the causal links between PM exposure and respiratory or cardiovascular disorders are well-established, emerging epidemiological evidence suggests a significant correlation between high ambient UPM levels and declining female fertility [7,8,9]. Recent studies have highlighted concerning trends, such as diminished ovarian reserve and increased risks of spontaneous abortion in populations residing in highly industrialized urban areas [8,9,10]. However, the detrimental effects of respiratory UPM on reproductive potential have not been clearly demonstrated.

The primary pathogenesis of UPM-induced injury involves the induction of a sustained inflammatory cascade and oxidative stress within the pulmonary microenvironment. Upon deposition in the distal airways and alveoli, UPM can trigger an innate immune response, leading to the release of pro-inflammatory mediators such as IL-6, IL-1β, and TNFα [2,11,12]. According to the ‘Lung-Gonad Axis’ hypothesis, these circulating cytokines and oxidative stressors may transcend the respiratory barrier to directly alter the sensitive ovarian microenvironment. Such systemic insults have been proposed to compromise oocyte quality and early embryonic development by overwhelming endogenous antioxidant defenses [13,14]. However, the specific toxicological threshold and the protective capacity of the female reproductive system against these respiratory-derived oxidative insults remain poorly defined. In this study, we comprehensively evaluated whether UPM-induced localized pulmonary insults induce molecular and functional changes in female reproduction in mice. While repeated intratracheal instillation (IT) of UPM induced localized pulmonary inflammation, no substantial alterations were detected in the maternal reproductive outcomes evaluated under the experimental conditions used in this study. Furthermore, direct exposure of preimplantation embryos to representative PAHs revealed that benzanthracene reduced blastocyst formation, whereas the other PAHs showed minimal effects on embryonic development under the experimental conditions tested. Collectively, our results suggest that the female reproductive tract is not substantially affected despite localized pulmonary inflammation induced by repeated UPM exposure.

## 2. Materials and Methods

### 2.1. Animals

All animal care and treatment procedures were approved by the Institutional Animal Care and Use Committee (IACUC) of CHA University (Approval No. IACUC 210068). Female ICR mice (4- and 6-week-old) and male ICR mice (9-week-old) were purchased from Orient Bio (Gapyeong, Gyeonggi, Republic of Korea). Mice were housed under temperature- and light-controlled conditions with the light on for 12 h daily and fed ad libitum. Six-week-old female mice were used for IT of UPM, whereas 4-week-old female mice were used to collect 2-cell embryos. Nine-week-old male mice were used for mating. Avertin was administered intraperitoneally (i.p) to induce general anesthesia, thereby minimizing animal movement and discomfort during the IT procedure and ensuring accurate delivery of UPM into the trachea.

### 2.2. UPM

Before IT, UPM (Standard Reference Material 1648a, Sigma-Aldrich, St. Louis, MO, USA) was dissolved in PBS and ultrasonicated for 15 min. SRM 1648a is a certified UPM reference material prepared by the National Institute of Standards and Technology from atmospheric PM collected in the St. Louis, Missouri, metropolitan area. It contains a complex mixture of chemical constituents, including PAHs, trace elements, and other organic compounds.

### 2.3. IT of UPM

Six-week-old female ICR mice were used to perform IT of UPM. For pulmonary analyses, 20 female mice were randomly assigned to five groups (NC, PBS, and UPM 40, 100, and 200 mg/kg; *n* = 4 per group). For embryo analyses, 12 female mice were randomly assigned to three groups (PBS, UPM 40 mg/kg, and UPM 200 mg/kg; *n* = 4 per group). For pregnancy and postnatal analyses, 24 female mice were randomly assigned to four groups (PBS and UPM 40, 100, and 200 mg/kg; *n* = 6 per group). Sample sizes were based on previous studies [15], and no animals or samples were excluded from the analyses. An IT method was modified from a previous study [16]. Briefly, after Avertin administration, mice were placed on a custom-made platform to facilitate tracheal intubation. Gel-loading tips (010-Q, Thermo Scientific QSP, Petaluma, CA, USA) were inserted into the trachea, and 30 μL of PBS or UPM suspension was instilled. The doses used in this study (40, 100, and 200 mg/kg) represent the amount of UPM administered per individual IT. The instillation procedure was repeated every 3 days for a total of 10 administrations. The untreated negative-control group received no anesthesia or IT and served as the baseline control for normal physiological conditions. In contrast, the PBS-treated group underwent the same anesthesia, intubation, and IT procedures without UPM exposure. This group served as the procedural control to account for the potential effects of these procedures.

### 2.4. Tissue Collection for Histological Examination, Hematoxylin and Eosin Staining, and Immunostaining

At 24 h after the final instillation, mice were euthanized by cervical dislocation, and the lungs, ovaries, and uteri were collected. Lung tissues were fixed in 4% paraformaldehyde (PFA), embedded in paraffin, and sectioned at a thickness of 5 μm. After deparaffinization in xylene and rehydration through a graded ethanol series, the sections were stained with hematoxylin and eosin according to standard protocols. Histological images were acquired using a light microscope (Carl Zeiss, Oberkochen, Germany).

Immunofluorescence (IF) staining was performed on paraffin-embedded lung tissue sections. Following deparaffinization and rehydration, antigen retrieval was carried out by heating the sections in a microwave for 20 min. After washing with PBS, nonspecific binding was blocked with Protein Block serum-free (Dako, Santa Clara, CA, USA) for 1 h at room temperature (RT). Sections were then incubated overnight at 4 °C with the appropriate primary antibodies (Supplementary Table S1). After washing with PBS, the sections were incubated with the corresponding Alexa Fluor-conjugated goat anti-rabbit or goat anti-rat secondary antibodies (1:200; Thermo Fisher Scientific, Waltham, MA, USA) for 1 h at RT. Nuclei were counterstained with DAPI (1:1000; Thermo Fisher Scientific). Fluorescence images were acquired at ×200 magnification using a fluorescence microscope (Carl Zeiss, Oberkochen, Germany) and analyzed with ZEN software (version 2 lite, Carl Zeiss).

### 2.5. RNA Preparation, RT-PCR, and Real-Time RT-PCR (qRT-PCR)

Total RNA was extracted from lung, ovary, and uterus tissues of UPM-exposed mice using TRIzol reagent (Invitrogen Life Technologies, Carlsbad, CA, USA), according to the manufacturer’s protocol. The first-strand cDNA was synthesized from 1 µg of total RNA using M–MLV reverse transcriptase (Promega, Madison, WI, USA) and RNasin Ribonuclease inhibitor (Promega). Synthesized cDNA (10 ng) was utilized for PCR with specific primers (Supplementary Table S2). qRT-PCR was performed using SYBR Green Dye (Bio-Rad, Waltham, MA, USA) to quantify gene expression. A standard curve of cycle thresholds from serial dilutions of a cDNA sample was generated to determine relative transcript levels, normalized to ribosomal protein L7 cDNA. All PCRs were performed in duplicate.

### 2.6. Cell Culture

RAW 264.7 cells (Korean Cell Line Bank, Seoul, Republic of Korea), a murine macrophage cell line, were maintained in 5% CO_2_ at 37 °C. DMEM high-glucose medium (Hyclone, Logan, UT, USA) supplemented with 10% fetal bovine serum (FBS, Gibco, Waltham, MA, USA) and 1% penicillin/streptomycin (1% P/S, Hyclone, USA) was used as the culture medium. 1.8 × 10^5^ cells were seeded in a 12-well plate, and several concentrations (12.5, 25, 50, and 100 μg/mL) of UPM were applied after 12 h of starvation.

### 2.7. Analyses of Fertilization Rate and Pregnancy Outcomes

To investigate the effects of intratracheal UPM exposure on fertilization and pregnancy outcomes, 24 female mice (*n* = 6 per group) were mated with fertile males 2 days after the final treatment without superovulation. The next morning, successful mating was confirmed by the presence of a vaginal plug, and this day was designated as gestation day 0. Time to conception, gestation length, and litter size were recorded after spontaneous delivery. Additionally, to evaluate postnatal development, body length at birth and body weight over the first 3 weeks after birth were recorded.

To collect 2-cell embryos, 12 female mice (*n* = 4 per group) were superovulated via i.p injection of CARD Hyperova (KYD-010-06-EX, Cosmo Bio Ltd., Carlsbad, CA, USA) followed by human chorionic gonadotropin (hCG; C1063-1VL, Sigma-Aldrich, USA). Oviduct flushing was performed at post-hCG 48 h using M2 medium (M7167-100ML, Sigma-Aldrich, USA) supplemented with 1% P/S to evaluate fertilization rate and the number of ovulated oocytes in each mouse. Two-cell embryos were cultured in KSOM medium (MR-121-D, Millipore, Burlington, MA, USA) until post-hCG 120 h. Blastocyst formation rate was assessed at post-hCG 120 h, after which the blastocysts were fixed in 4% PFA for subsequent staining.

### 2.8. Embryo Staining and Quality Examination

Blastocysts were fixed in 4% PFA for 1 h at 4 °C and washed with PBS containing 0.1% polyvinyl alcohol. After permeabilization with 0.2% Triton X-100 in PBS, embryos were blocked with Protein Block Serum for 1 h at RT. The blastocysts were then incubated overnight at 4 °C with an anti-OCT4 primary antibody (1:100, 611203, BD Biosciences, Franklin Lakes, NJ, USA). After washing, embryos were incubated with Alexa Fluor 594-conjugated secondary antibody (1:200, Jackson) for 1 h at RT. Nuclei were counterstained with DAPI (1:1000, 62248, Thermo Fisher Scientific). Fluorescence images were acquired using a fluorescence microscope (Carl Zeiss, Germany) and analyzed with ZEN software (Carl Zeiss). The total cell number and the inner cell mass (ICM)-to-trophectoderm (TE) ratio were determined based on OCT4 and DAPI staining.

### 2.9. Preparation of PAHs for Embryo Culture

Benzanthracene (B2209-500MG), 9,10-Phenanthrenequinone (156507-5G), 9-fluorenone (F1506-100G-A), and Perinaphthenone (P10801-1G) were purchased from Sigma-Aldrich (St Louis, MO, USA). Each PAH was initially dissolved in DMSO and diluted to 1/1000 in KSOM. The highest working concentrations of each material: Benzanthracene, 20 μM; 9,10-phenanthrenequinone, 30 μM; 9-fluorenone, 500 μM; Perinaphthenone, 250 μM. Fresh 2-cell embryos were retrieved from 4-week-old ICR female mice by superovulation and cultured in KSOM with one of the PAHs used in this study, from 48 to 120 h post-hCG. The morphology and developmental progression of 2-cell embryos were evaluated. The total cell number and the ICM-to-TE ratio of the blastocyst were examined as previously described [15].

### 2.10. Statistical Analysis

All statistical analyses were performed using GraphPad Prism (version 8.2, GraphPad Software, San Diego, CA, USA). Comparisons among three or more groups were performed using one-way analysis of variance (ANOVA) followed by Dunnett’s multiple comparisons test. For analyses of the number of ovulated oocytes and fertilization rates, the individual dam was considered the independent biological unit. Following assessment of fertilization, embryos from four dams within each treatment group were pooled for subsequent in vitro culture, and dam-of-origin information was not retained. These pooled embryo data were therefore evaluated descriptively without inferential statistical analysis. For body length and longitudinal body-weight analyses, measurements from pups within each litter were averaged, with the litter/dam considered the independent biological unit. Body length was analyzed using one-way ANOVA followed by Dunnett’s multiple comparisons test, whereas longitudinal body-weight data were analyzed using two-way repeated-measures ANOVA with Geisser–Greenhouse correction, followed by Dunnett’s multiple comparisons test. Data are presented as the mean ± standard deviation (SD) or median, as appropriate. A *p* value < 0.05 was considered statistically significant.

## 3. Results

### 3.1. IT of UPM Induces Localized Pulmonary Inflammation and Molecular Fibrotic Signaling

**Figure 1:**
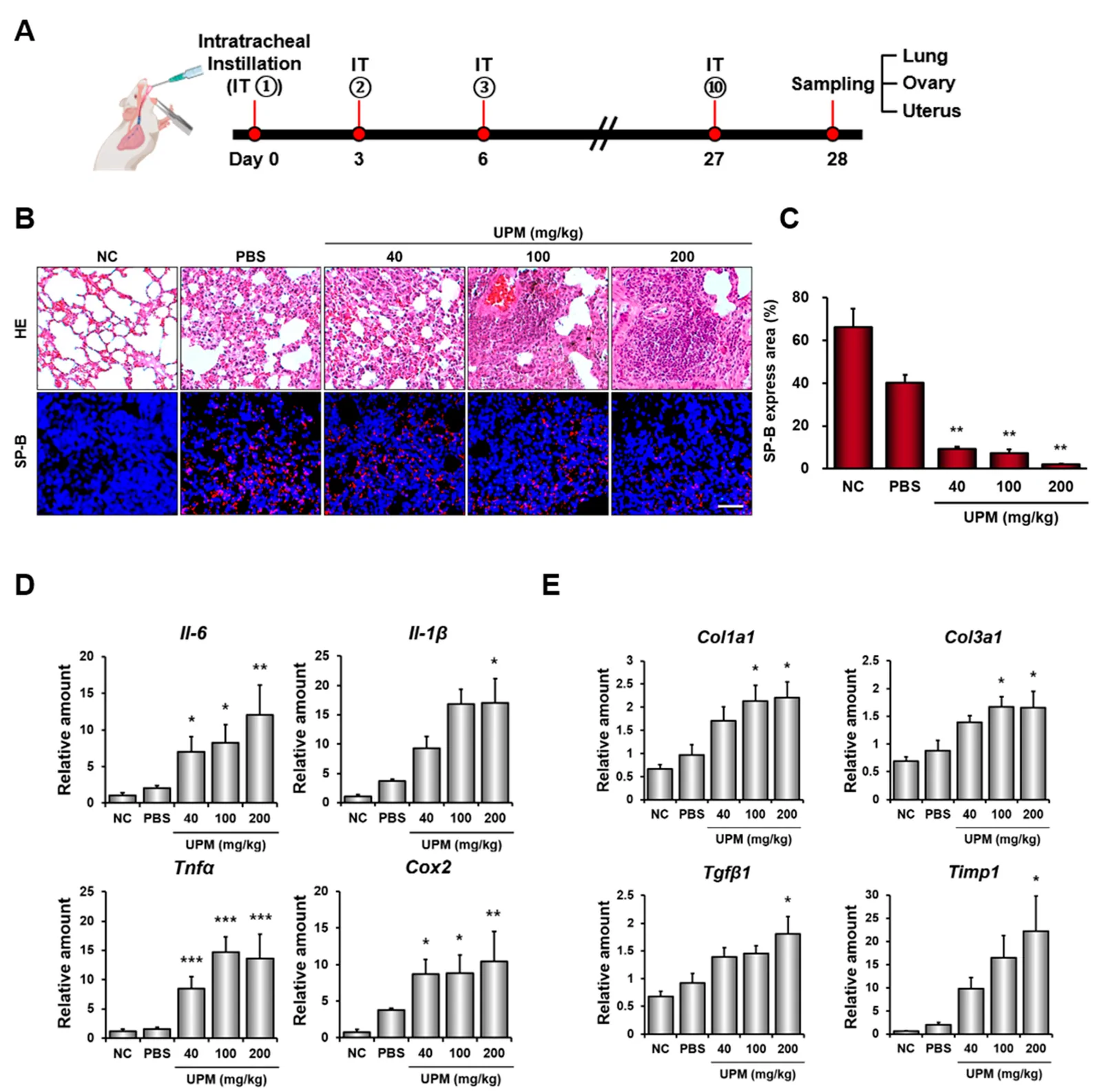
IT of UPM induces histopathological changes and molecular inflammatory/fibrotic signaling in mouse lungs. (**A**) Experimental schematic for UPM exposure. UPM was administered via IT every 3 days for a total of 10 times over one month. (**B**) Representative images of Hematoxylin and Eosin staining and SP-B IF staining in lungs exposed to UPM. (*n* = 4 mice per group). Scale bar = 50 μm. (**C**) Quantification of SP-B-positive area after treatment with increasing concentrations of UPM. (**D**,**E**) Relative mRNA expression levels of proinflammatory cytokines (*Il-6*, *Il-1β*, *Tnfα*, and *Cox2*) and fibrosis markers (*Col1a1*, *Col3a1*, *Tgfβ1*, and *Timp1*) in lung tissues were quantified by qRT-PCR. Data represent the mean ± SD (*n* = 4 mice per group). Statistical significance was determined by one-way ANOVA followed by Dunnett’s multiple comparisons test (* *p* < 0.05, ** *p* < 0.01, *** *p* < 0.001 vs. PBS group).

To investigate the systemic impact of air pollution via the respiratory route, mice were subjected to repeated IT of UPM for one month (Figure 1A). Histopathological analysis of lung tissues by HE staining revealed dose-dependent thickening of the alveolar septa, whereas IF staining demonstrated a dose-dependent decrease in SP-B expression (Figure 1B,C). The significant recruitment of CD45+, F4/80+, and Ly6G+ immune cells confirmed successful UPM delivery and subsequent pulmonary insult characterized by increased inflammatory cell infiltration (Supplementary Figure S1). Molecular analysis further confirmed a robust inflammatory response, with a significant upregulation of pro-inflammatory cytokines (*Il-6*, *Il-1β*, *Tnfα*, and *Cox2*) and fibrotic signaling markers (*Col1a1*, *Col3a1*, *Tgfβ1*, and *Timp1*) in the lung parenchyma (Figure 1D,E). Given the marked inflammatory cell infiltration observed in the lungs following UPM exposure, we next examined whether UPM directly activates an inflammatory response in macrophages. RAW 264.7 cells, a mouse macrophage cell line, were treated with increasing concentrations of UPM (0, 12.5, 25, 50, 100 μg/mL) for 24 h. UPM treatment significantly increased the mRNA expression of the pro-inflammatory cytokines in a dose-dependent manner, indicating that UPM directly elicits an inflammatory response in macrophages (Supplementary Figure S2).

### 3.2. Pulmonary UPM Exposure Does Not Compromise Oocyte Competence and Subsequent Early Embryo Development

**Figure 2:**
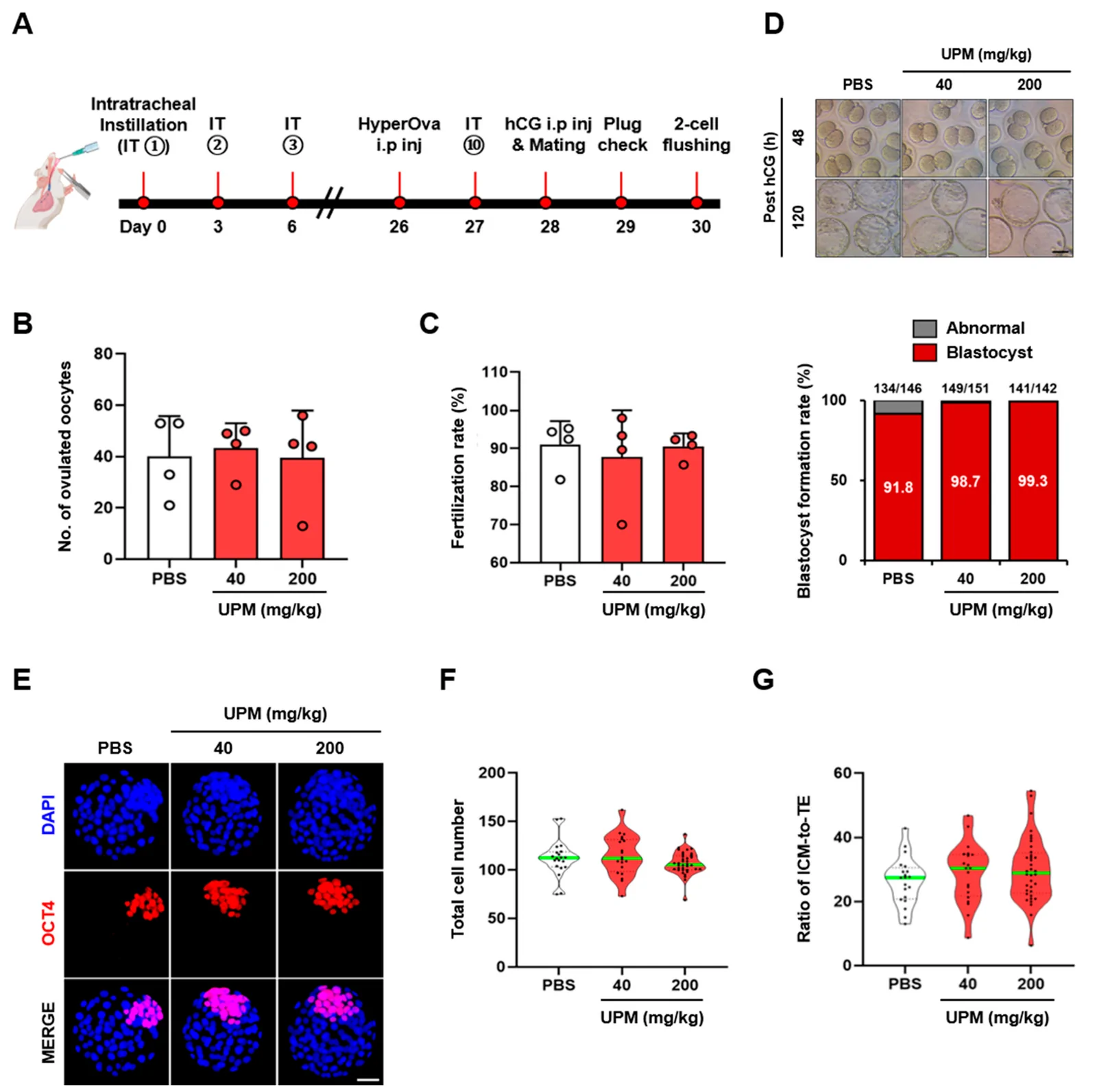
Quality examination of preimplantation embryos from UPM-exposed mice. (**A**) Experimental schematic of UPM exposure, superovulation, mating, and collection of 2-cell embryos. (**B**) Number of ovulated oocytes per dam (*n* = 4 dams per group). ((**C**), left) Fertilization rates per dam (n = 4 dams per group). Data in (**B**) and ((**C**), left) were analyzed by one-way ANOVA followed by Dunnett’s multiple comparisons test. ((**C**), right) Blastocyst formation rates of embryos pooled from four dams per group. (**D**) Representative images of 2-cell embryos and blastocysts from PBS- and UPM-exposed mice. (*n* = 4 dams per group). Scale bar = 50 μm. (**E**) Representative IF images of OCT4 (red) and DAPI (blue) staining in blastocysts at post-hCG 120 h. Scale bar = 50 μm. (**F**,**G**) Evaluation of total cell number and the ICM-to-TE ratio in blastocysts derived from the pooled embryo samples. Each dot represents an individual blastocyst. Green lines indicate the median, and dotted lines indicate the first and third quartiles (25th and 75th percentiles, respectively). Dam-level replication was not retained for ((**C**), right), and (**F**,**G**) because embryos were pooled before subsequent culture.

We then evaluated whether UPM-induced pulmonary injury affected oocyte fertilization competence and subsequent preimplantation embryo development in mice (Figure 2A). There were no significant differences in the number of recovered oocytes or fertilization rates between the control group and the low-dose (40 mg/kg) or high-dose (200 mg/kg) UPM-exposed groups (Figure 2B,C). For subsequent developmental assessment, embryos from different dams within each group were pooled, precluding dam-level statistical comparisons. Blastocyst formation was observed at similar rates across the pooled groups (Figure 2C,D). IF staining for OCT4, a marker of the ICM, also showed similar distributions of total cell number and ICM-to-TE ratio across the pooled groups (Figure 2E–G). Furthermore, RT-PCR analysis showed that expression of inflammatory marker genes was not detectable in the ovaries or uteri (Supplementary Figure S3).

### 3.3. IT of UPM Before Pregnancy Does Not Affect Maternal Pregnancy Outcomes and Subsequent Postnatal Development

**Figure 3:**
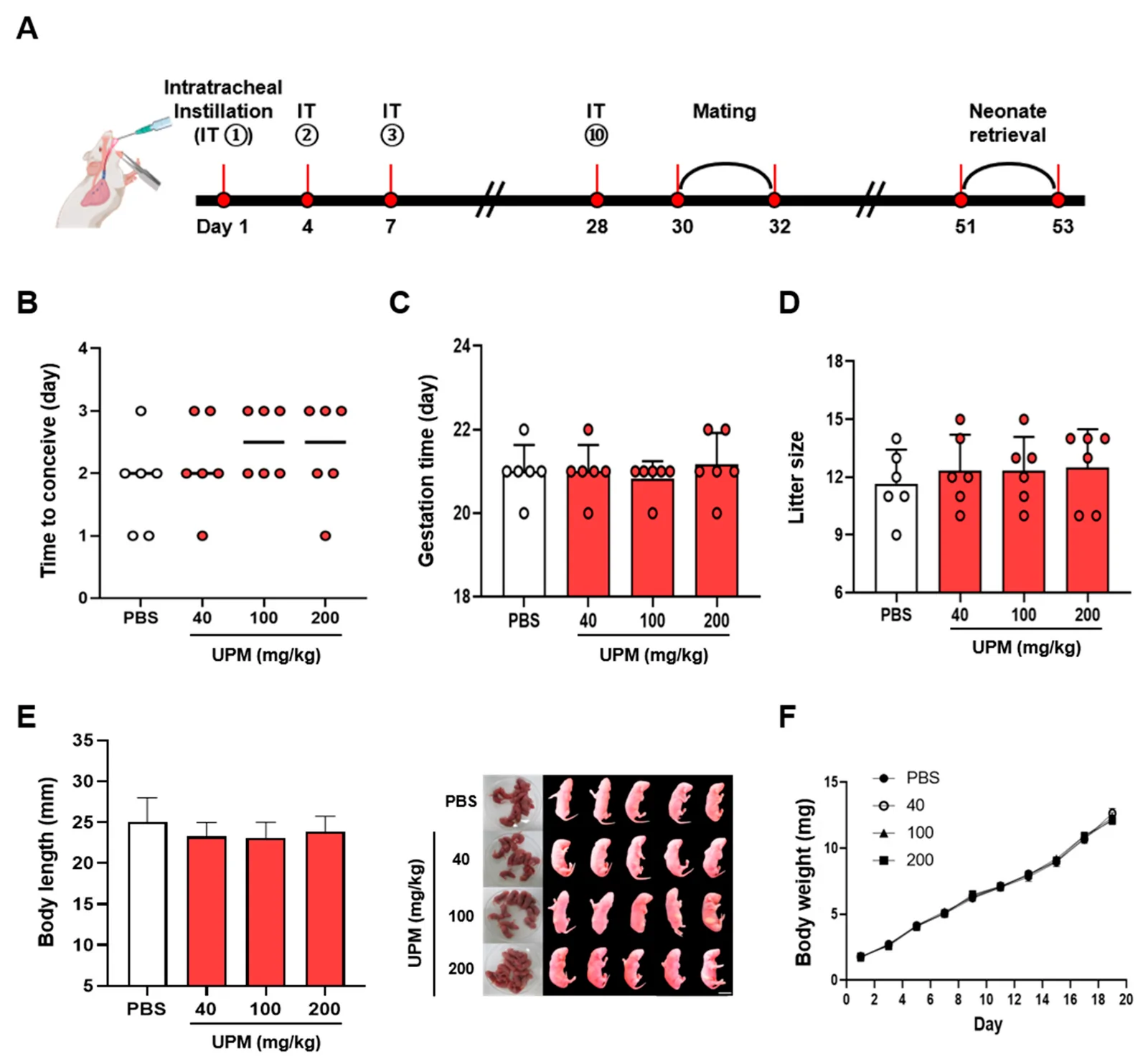
Effects of intratracheally instilled UPM on maternal pregnancy and postnatal development of pups. (**A**) Experimental scheme illustrating the timeline from the final UPM instillation to successful mating. (**B**–**D**) Quantitative analysis of time to conceive, gestation time, and litter size in mice following UPM exposure (*n* = 6 dams per group). (**E**,**F**) Body length and longitudinal body weight of pups, analyzed at the litter/dam level. Body length (**E**) was analyzed by one-way ANOVA followed by Dunnett’s multiple comparisons test, and longitudinal body weight (**F**) by two-way repeated-measures ANOVA with Geisser–Greenhouse correction followed by Dunnett’s multiple comparisons test. Scale bar = 1 cm.

To further evaluate the potential impact of respiratory UPM exposure on pregnancy outcomes, UPM-exposed female mice were mated with fertile ICR males two days after the final instillation (Figure 3A). Respiratory UPM exposure did not interfere with reproductive parameters, including time to conceive (Figure 3B), gestation duration (Figure 3C), and litter size (Figure 3D). In addition, the average body length at birth (Figure 3E) and subsequent body weight during the postnatal period (Figure 3F) showed no significant impairments across all dosage groups.

### 3.4. Direct Exposure to UPM and PAHs Does Not Interfere with Preimplantation Embryo Development In Vitro

**Figure 4:**
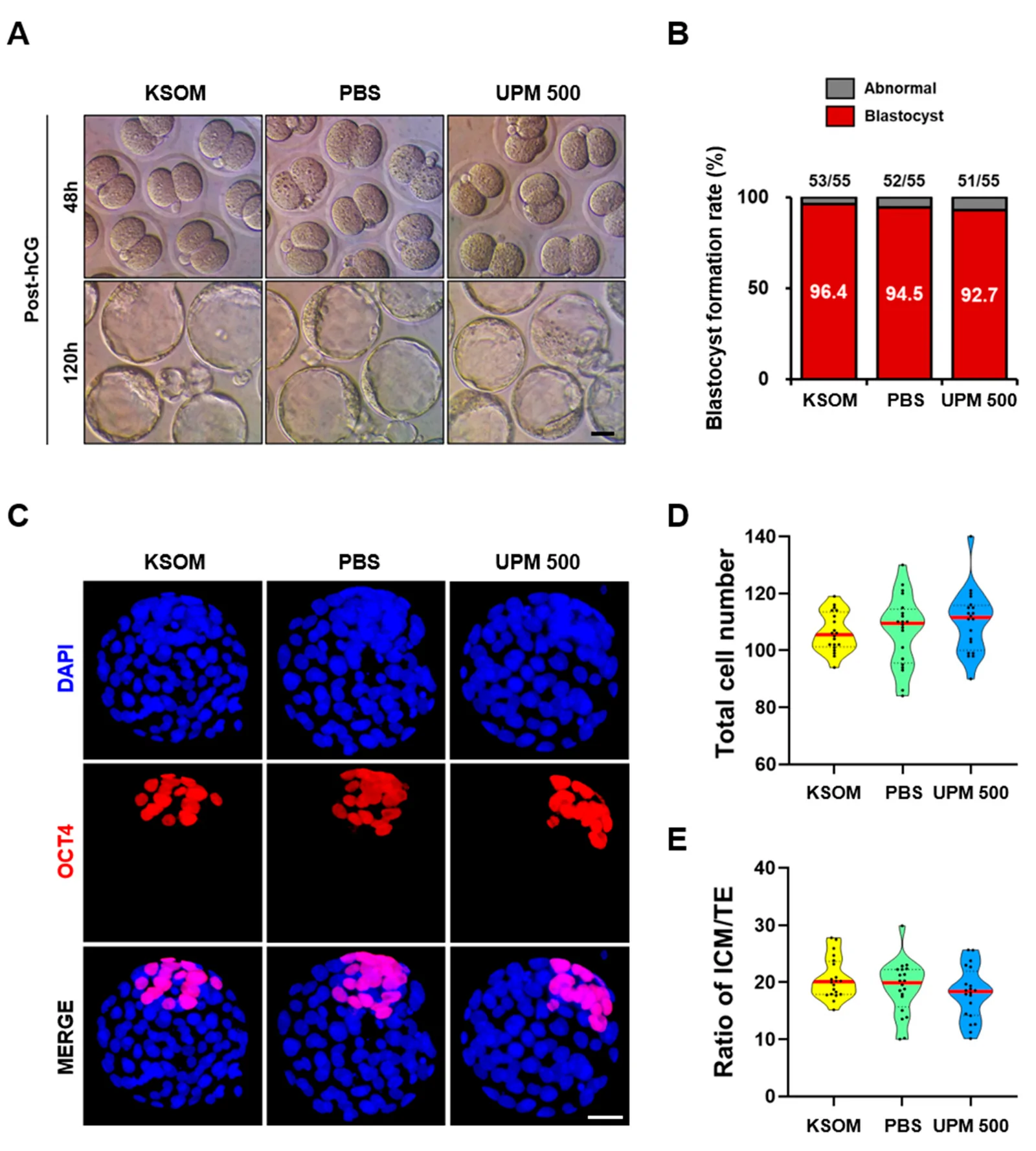
Effects of direct UPM exposure on preimplantation embryo development in vitro. (**A**) Representative images of 2-cell embryos and blastocysts cultured with KSOM, PBS, and UPM 500 μg/mL. (*n* = 55 embryos per group). Scale bar = 50 μm. (**B**) Blastocyst formation rates were determined using 2-cell embryos cultured in vitro for 120 h post-hCG. Statistical significance was determined using the chi-square test. (**C**) Representative IF images of OCT4 (red) and DAPI (blue) staining in blastocysts. Scale bar = 50 μm. (**D**,**E**) Quantitative analysis of total cell number and the ICM-to-TE ratio. Each dot represents an individual blastocyst. Red lines indicate the median, and dotted lines indicate the first and third quartiles (25th and 75th percentiles, respectively). Statistical significance was determined by one-way ANOVA followed by Dunnett’s multiple comparisons test.

Because repeated intratracheal UPM exposure induced pulmonary inflammation without apparent adverse reproductive effects in vivo, we next examined whether direct exposure of preimplantation embryos to relatively high concentrations of UPM and representative PAHs affected embryonic development in vitro. Two-cell embryos were retrieved from 4-week-old superovulated ICR female mice and cultured in KSOM media supplemented with UPM. The morphology of embryos observed from 48 to 120 h post-hCG appeared normal, and the blastocyst formation rate of embryos was similar across all groups (Figure 4A,B). The total cell number and the ICM-to-TE ratio did not differ significantly among the groups (Figure 4C–E).

**Figure 5:**
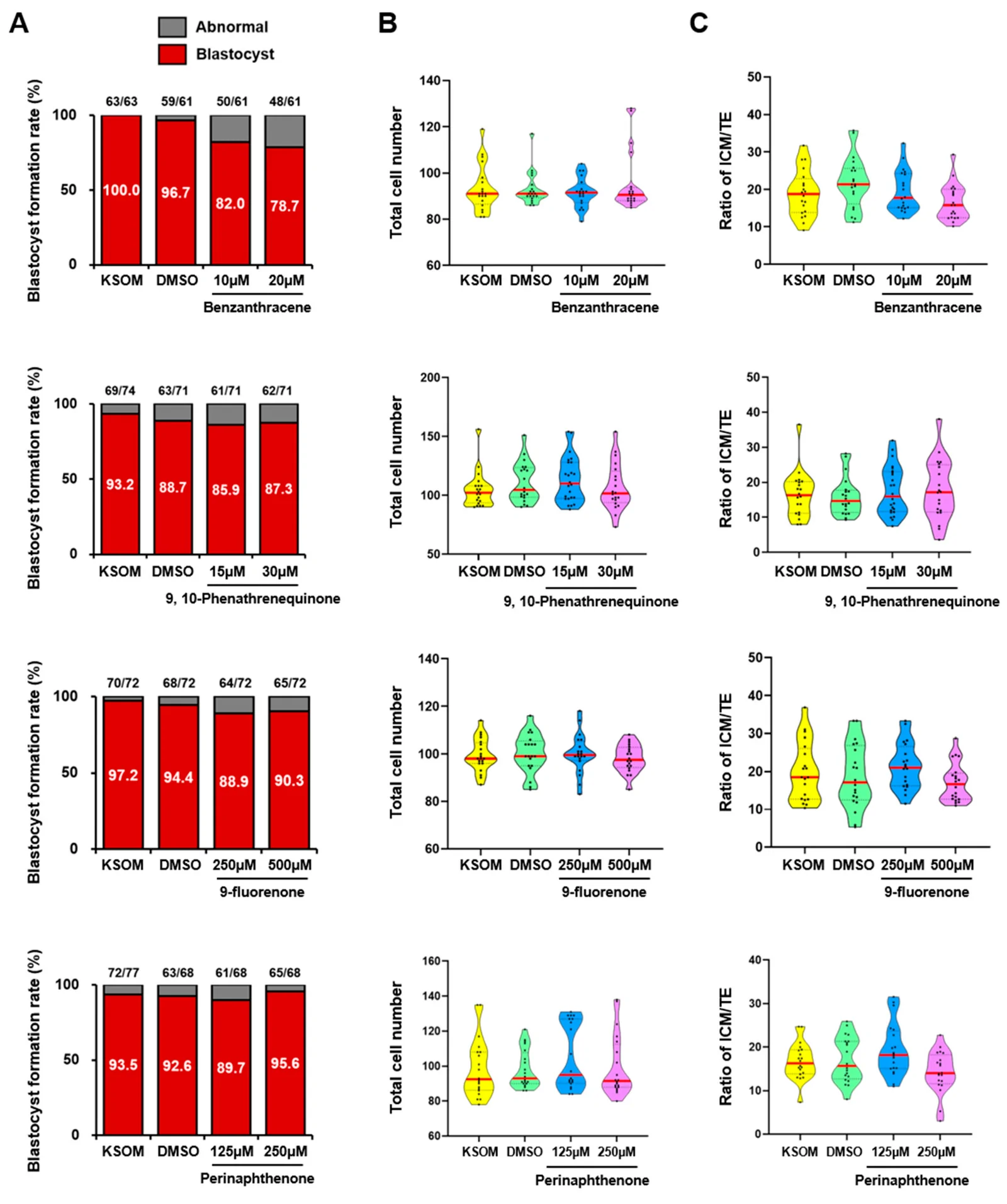
Selective embryotoxicity of UPM-associated PAH components during in vitro culture. (**A**) Blastocyst formation rates were determined using 2-cell embryos cultured in KSOM supplemented with four major PAH components (Benzanthracene, 9,10-phenanthrenequinone, 9-fluorenone, and Perinaphthenone). (**B**,**C**) Quantitative analysis of total cell number and ICM-to-TE ratio. Each dot represents an individual blastocyst. Red lines indicate the median, and dotted lines indicate the first and third quartiles (25th and 75th percentiles, respectively). Statistical significance was determined by one-way ANOVA followed by Dunnett’s multiple comparisons test.

Given that UPM contains various PAHs, we evaluated whether representative PAHs present in UPM affect preimplantation embryo development in vitro. Among the PAHs tested, Benzanthracene (BaA) significantly inhibited blastocyst development of 2-cell embryos in a dose-dependent manner. In contrast, the oxygenated PAHs (OPAHs) 9,10-phenanthrenequinone, 9-fluorenone, and Perinaphthenone did not significantly impair blastocyst development (Figure 5A). Blastocysts derived from 2-cell embryos cultured with either BaA or OPAHs showed comparable total cell numbers and ICM-to-TE ratios to those of the control embryos (KSOM and DMSO groups) (Figure 5B,C).

## 4. Discussion

The present study provides a comprehensive evaluation of female reproductive outcomes following localized respiratory exposure. The induction of pulmonary inflammation observed in our IT model is highly consistent with the established literature on PM-induced respiratory toxicity [12]. The significant increase in the infiltration of CD45+, F4/80+, and Ly6G+ immune cells, coupled with the upregulation of proinflammatory cytokines such as *Il-6*, *Il-1β*, and *Tnfα* (Figure 1), confirms that the lung tissues were under substantial inflammatory stress [17]. Furthermore, the reduced SP-B expression and the upregulation of fibrosis markers (*Col1a1* and *Tgfβ1*) align with recent studies suggesting that PM exposure disrupts alveolar homeostasis and initiates the early stages of pulmonary remodeling [12,18]. Nevertheless, no substantial alterations were detected in the maternal reproductive outcomes evaluated under the exposure conditions used in this study. These findings are particularly significant given the increasing epidemiological evidence linking ambient air pollution to impaired fertility [7,19].

Cytokines released from the lung into the systemic circulation are expected to disrupt the hypothalamus-pituitary-gonad axis or directly impair the ovarian microenvironment [19,20]. Although recent epidemiological studies have characterized PM as a significant risk factor for adverse pregnancy outcomes [8,10,21], the reproductive outcomes examined were not substantially altered following repeated maternal exposure to UPM in mice. Specifically, our data show that pre-gestational exposure to UPM did not significantly alter the time to conceive, gestation length, or neonatal growth trajectories (Figure 3). Qualitative RT-PCR analysis showed no apparent increase in inflammatory-marker expression in the ovaries and uteri following UPM exposure (Supplementary Figure S3). Moreover, ovarian functions, including oocyte recovery rates and fertilization rates, were not significantly compromised. These findings contrast with several recent reports documenting follicular atresia and reduced endometrial receptivity following PM exposure [22,23]. Notably, Park et al. recently reported endometrial inflammation and impaired fertility using the same reference material (SRM 1648a) [24]. This discrepancy may be attributable to differences in experimental design, including the mouse strain (ICR vs. C57BL/6), route of exposure (intratracheal vs. intranasal administration), and the reproductive endpoints evaluated. In addition, discrepancies between our findings and studies using ambient PM2.5 may also reflect intrinsic physicochemical differences between SRM 1648a and contemporary PM2.5 [22,23]. We deliberately selected SRM 1648a to ensure methodological reproducibility and a standardized chemical baseline, which is a widely accepted practice in PM toxicology. SRM 1648a represents a time-integrated atmospheric sample collected in 1976–1977, featuring a mean particle diameter of approximately 5.85 µm [25]. These characteristics skew its profile more toward the coarse PM fraction (PM10) than contemporary fine PM2.5 or ultrafine particles. Because smaller particles possess a higher specific surface area and a greater capacity to translocate across biological barriers [26], the relatively large particle size of SRM 1648a is expected to limit the systemic bioavailability of these particles to distant organs, including the gonads [27].

Recent studies increasingly support the view that the reproductive risks of air pollution—such as impaired fertility, poor oocyte/sperm quality, and adverse birth outcomes—are primarily driven by specific chemical constituents rather than the total PM mass (like overall PM2.5 or PM10 levels), highlighting the limitation of relying solely on crude regulatory metrics [28]. We also observed no significant alterations in oocyte recovery or fertilization following respiratory exposure to SRM 1648a before ovulation induction (Figure 2). Moreover, the observed blastocyst formation rates and blastocyst quality were similar across the pooled groups. However, the loss of dam-of-origin information after pooling embryos from different dams represents a limitation of the present study, particularly for the analyses of blastocyst formation, total cell number, and ICM-to-TE ratio. Toxicological evidence indicates that specific components, particularly PAHs, heavy metals such as Pb, Cd, and Cu, and secondary inorganic aerosols, dictate the particle’s capacity to cross biological barriers and compromise reproductive health [29,30]. PAHs act as potent endocrine-disrupting chemicals that bind to the aryl hydrocarbon receptor (AhR) [31], disrupting the Hypothalamus-Pituitary-Gonad axis, impairing steroidogenesis, and inducing follicular atresia or abnormal sperm morphology [32,33,34]. While BaA represents a major parent PAH in urban dust, atmospheric aging transforms such compounds into OPAHs. For instance, 9,10-phenanthrenequinone, 9-fluorenone, and Perinaphthenone in this study are OPAHs from phenanthrene, fluorene, and phenalene, respectively, and serve as critical indicators of the real-world oxidative potential of ambient fine PM. Despite the reported higher toxicity of OPAHs than their parent compounds in various cell types, the three OPAHs examined in this study did not significantly impair preimplantation embryo development (Figure 5). Further studies are needed to elucidate how preimplantation mouse embryos develop normally under conditions with harmful OPAHs in vitro.

Previous studies have demonstrated that certain high-molecular-weight PAHs, most notably benzo[a]pyrene (BaP), induce the production of reactive oxygen species, leading to increased DNA damage and apoptosis, and compromised genomic stability in mouse preimplantation embryos [35]. In contrast, our current findings reveal that the four PAHs—BaA and OPAHs, 9,10-phenanthrenequinone, 9-fluorenone, and Perinaphthenone— did not induce severe developmental abnormalities overall, although benzanthracene reduced blastocyst formation (Figure 5). This discrepancy may reflect differential activation of AhR. BaP-induced embryotoxicity typically requires canonical AhR signaling, in which high-affinity ligands induce the transcriptional upregulation of cytochrome P450 enzymes (e.g., CYP1A1 and CYP1B1), thereby converting parent compounds into highly reactive, mutagenic epoxide intermediates [36]. Compared to the five-ring structure of BaP, the four-ring BaA exhibits a lower AhR binding affinity due to its distinct spatial geometry and suboptimal fit within the receptor’s ligand-binding pocket [37]. OPAHs exhibit a much lower binding affinity than BaP [38], suggesting that OPAHs in this study may lack the capacity to activate this specific receptor-mediated toxic signaling at the tested concentrations. This differential activation of AhR likely explains why BaA and OPAHs induced only a moderate reduction in blastocyst development rate and no apparent damage to preimplantation mouse embryos, respectively (Figure 5).

## 5. Conclusions

Our study demonstrates that marked pulmonary inflammation induced by repeated short-term UPM exposure was not accompanied by substantial alterations in the female reproductive outcomes examined under the exposure conditions used. These findings suggest that future clinical assessments of pollution-related fertility issues should consider not only PM concentration and particle size but also the duration of exposure and the potential bioavailability of high-risk components such as BaP. This research provides a foundation for understanding the relationship between pulmonary UPM exposure and female reproductive outcomes and highlights the need for nuanced risk assessment models in environmental toxicology.

## Data Availability

The data that support the findings of this study are available from the corresponding author upon reasonable request.

## Supplementary Materials

The following supporting information can be downloaded at: https://www.mdpi.com/article/10.3390/cells15171522/s1, Figure S1: Histopathological and IF analyses of lung tissues following UPM exposure. (A–C) Representative IF images and quantitative analysis of the positive staining area for the immune cell markers CD45 (leukocytes), F4/80 (macrophages), and Ly6G (neutrophils). Scale bar = 50 μm; Figure S2: Effects of UPM on mouse macrophage cell line. (A) Experimental protocol for UPM treatment of RAW 264.7 cells. Cells were serum-starved for 12 h and then treated with UPM (0, 12.5, 25, 50, and 100 μg/mL) for 24 h. (B) qRT-PCR analysis of the relative mRNA expression levels of the proinflammatory cytokines. Gene expression levels were normalized to the internal control and are presented relative to the control group. Data are presented as the mean ± SEM. Statistical significance was determined by one-way ANOVA followed by Dunnett’s multiple comparisons test (* *p* < 0.05; ** *p* < 0.01; *** *p* < 0.001). (C) Representative bright-field images of RAW 264.7 cells treated with increasing concentrations of UPM for 24 h, demonstrating dose-dependent morphological changes; Figure S3: RT-PCR analyses for mRNA expression levels of pro-inflammatory cytokines in ovaries (A) and uteri (B). RAW 264.7 cells treated with LPS (100 ng/mL) for 24 h were used as a positive control (PC); Figure S4: IF staining of OCT4 in blastocysts cultured with PAHs in vitro. (A–D) IF images showing OCT4 (red) and DAPI (blue) staining in blastocysts cultured with four major PAH components (Benzanthracene, 9,10-phenanthrenequinone, 9-fluorenone, and Perinaphthenone). Scale bar = 50 μm. Supplementary Table S1. List of primary antibodies used for IF analysis in this study. Supplementary Table S2. Primer sequences used for quantitative qRT-PCR analysis in this study. Supplementary Table S3. Results of Dunnett’s multiple comparisons test for total cell number and ICM-to-TE ratio following in vitro UPM exposure. Mean differences, 95% confidence intervals (CIs), adjusted *p* values, and statistical significance are presented for each comparison. Supplementary Table S4. Results of Dunnett’s multiple comparisons test for total cell number and ICM-to-TE ratio following exposure to representative PAHs. In addition to adjusted *p* values, mean differences and 95% CIs obtained from Dunnett’s multiple comparisons test are provided.
